# Calpainopathy: Description of a Novel Mutation and Clinical Presentation with Early Severe Contractures

**DOI:** 10.3390/genes11020129

**Published:** 2020-01-25

**Authors:** Iván Landires, Virginia Núñez-Samudio, Julián Fernandez, Cesar Sarria, Víctor Villareal, Fernando Córdoba, Giovanni Apráez-Ippolito, Samuel Martínez, Oscar M. Vidal, Jorge I. Vélez, Mauricio Arcos-Holzinger, Sergio Landires, Mauricio Arcos-Burgos

**Affiliations:** 1 Instituto de Ciencias Médicas, Las Tablas, Los Santos 0710, Panama; ivanlandires@yahoo.es (I.L.); virysamudio@yahoo.es (V.N.-S.); oscararcos98@gmail.com (M.A.-H.); 2 Fundación José María Delgado-Paredes para Promover la Investigación en Medicina, Popayán, Colombia; juanfeco73@hotmail.com (J.F.); cesarria@hotmail.com (C.S.); victorvilla1973@hotmail.com (V.V.); cordoballanos@gmail.com (F.C.); giovanniapraez@gmail.com (G.A.-I.); smb2001@hotmail.com (S.M.); 3 Centro Regional Universitario de Azuero, CRUA, Universidad de Panamá, Chitré 0601, Herrera, Panama; 4 Universidad del Norte, Barranquilla 080008, Colombia; oorjuela@uninorte.edu.co (O.M.V.); jvelezv@uninorte.edu.co (J.I.V.); 5 Centro Integral de Radiodiagnóstico, Chitré 0601, Herrera, Panama; drsergiolandires@gmail.com; 6 Grupo de Investigación en Psiquiatría (GIPSI), Departamento de Psiquiatría, Instituto de Investigaciones Médicas, Facultad de Medicina, Universidad de Antioquia, Medellín 050010, Colombia

**Keywords:** calpain 3-related, limb-girdle muscular dystrophy type r1, calpain gene, calpainopathy, Emery–Dreifuss-like syndrome, novel mutation, deletion, clinical presentation, Amerindian populations, founder effect

## Abstract

Presented here are five members of a family that was ascertained from an isolated, consanguineous, indigenous Amerindian community in Colombia that was affected with calpain 3-related, limb-girdle muscular dystrophy type R1. These patients are homozygous for a unique and novel deletion of four bases (TGCC) in exon 3 of the calpain 3 gene (*CAPN3*) (NM_000070.2; NP_000061.1) (g.409_412del). The mutation site occurs at the CysPc protein domain, triggering a modified truncated protein structure and affecting motifs within the calpain-like thiol protease family (peptidase family C2) region. The patients reported here developed a very severe phenotype with primary contractures, spinal rigidity in the early stages of the disease, and bilateral talipes equinovarus (clubfoot) in the most affected patients who had the selective involvement of their extremities’ distal muscles in a way that resembled Emery–Dreifuss syndrome. We recommend mandatory screening for calpainopathy in all patients with an Emery–Dreifuss-like syndrome or those presenting a non-congenital illness with primary contractures and who, because of other data, are suspected of having muscular dystrophy.

## 1. Introduction

In 1954, John Walton and Frederick Nattrass described limb-girdle muscular dystrophy (OMIM: 253600) as muscular weakening with an onset during the first three decades of life, which follows an autosomal recessive model of inheritance, and which affects either the shoulder or pelvic girdle but without the compromise of both the facial muscles and the pseudohypertrophy of the calves [1,2,3].

In 1991, by studying families from “La Reunion Island,” Jacques Beckmann linked this phenotype to a minimal critical region (MCR) that is harbored in chromosome 15q [4]. Later, it was found that mutations that are harbored in this 15q MCR in the *Homo sapiens CAPN3* gene—which encodes the proteolytic enzyme calpain-3—are the cause of this phenotype (named muscular dystrophy, limb-girdle, type 2a; muscular dystrophy, limb-girdle, type 2; Lgmd2, muscular dystrophy, pelvofemoral, Leyden-Moebius muscular dystrophy, calpainopathy, limb-girdle muscular dystrophy type 2a, Lgmd2a, and calpain 3-related, limb-girdle muscular dystrophy type R1, Lgmdr1) [5]. 

Additional studies of this phenotype were carried out in genetically isolated, endogamous communities from the Basque Country region [6], the Amish Community [7], Turkey [8], and the Italian Alps [9]. The term calpainopathy was coined to identify the cluster of signs and symptoms that define the phenotype that originated as consequence of pathogenic mutations that are harbored in the *CAPN3* gene. Furthermore, Pollitt et al. (2001) and Mercuri et al. (2005) independently reported on a variant of clinical expression of calpainopathy that showed spinal rigidity and early primary contractures [10,11]. 

In this report, we characterize an indigenous Amerindian, consanguineous family by segregating a novel deletion of four bases (TGCC) in exon 3 of the *calpain 3* gene (*CAPN3*) (NM_000070.2; NP_000061.1) (g.409_412del), clustering a phenotype with early severe contractures.

We are sure that this severe clinical presentation will be important to outline the calpainopathy phenotype, which, at the end, will complement the differential diagnosis and definition of correlations between mutations (genotype), clinical presentation (molecular phenotype), and the natural history of this devastating disease.

## 2. Materials and Methods

### 2.1. Ethics Statement, Consent, and Permissions

This study was conducted in accordance with the Declaration of Helsinki and was approved by the Institutional Review Board of the Antioquia University, Colombia. Written informed consent was obtained from each participant and their parents.

### 2.2. Subjects

**Figure 1:**
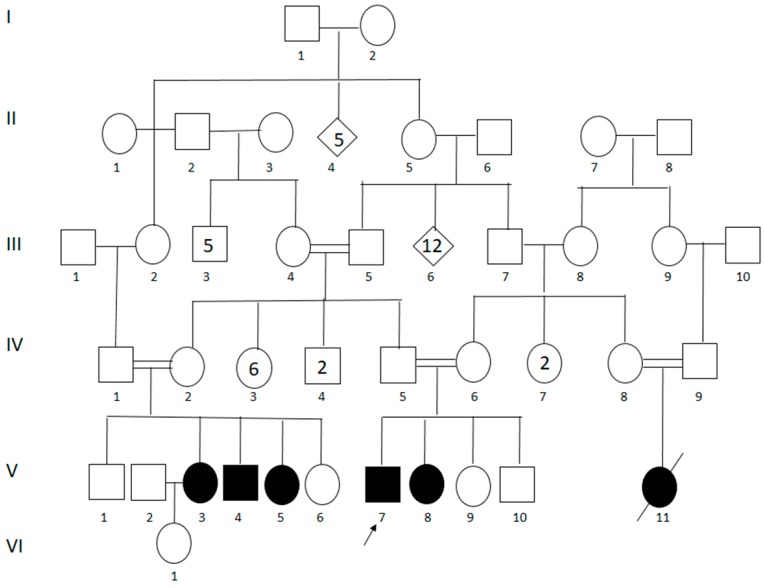
Genealogical tree revealing consanguinity. V3: case 1; V4: case 2; V5: case 3; V7: case 4; and V8: case 5. Numbers inside of the symbols represent the sum of the individuals with this standard representation.

Originally from “El Carmen-Piendamo,” State of Cauca, in the Southwestern Andean region of Colombia, the patients were ascertained at the “Hospital Universitario San José de Popayán.” The family belonged to a geographically and culturally isolated community of ~1200 aboriginal Amerindians from the “Paez” tribe, where the affected proband had originally been diagnosed with limb-girdle muscular dystrophy after presenting signs and symptoms that were compatible with the disease. Later, four more cases were described, and a sixth member was said to have been affected by the same syndrome but had died at the age of fourteen of unknown causes (Figure 1).

### 2.3. Clinical Assessment

The diagnosis was based on parameters of the clinical–neurological examination and studies of electrophysiology, histology, biochemistry, and genetics. Diagnostic criteria were based on those that were proposed by the 229th European Neuromuscular Centre Workshop and which have changed the nomenclature on calpainopathy from calpain 3-related LGMD2A to LGMDR1 [12].

A continuous 4-year evaluation of the five living members of the family (two males and three females) was performed. The protocol included a detailed examination of muscular strength by using the guidelines of the Medical Research Council (1943) [13] and a functional stratification of muscular dystrophies ranging from stage I to stage VII in accordance with the scale proposed by Gardner-Merwin and Walton (1974) [14]. Photographs were taken of each patient. The analyses, done on all of the patients with their prior consent, included: serum creatine kinase (CK) levels, electromyography, sensory and motor neuro-conduction velocities, a histopathological study, an electrocardiogram, a bi-dimensional echocardiogram, chest x-rays, spirometry, psychological evaluations (Wechsler scale), and molecular genetic studies. Computerized axial tomography, used to examine the extent of muscular involvement of the lower limbs, was done in 2 patients who gave their consent. For four of the patients with different degrees of symptoms severity, a biopsy of the deltoid muscle was taken by using the “mini-open” procedure. The histopathological studies were done with hematoxylin–eosin and with Mason’s trichromic stains. Genealogical trees were diagramed with the help of the patients and their families (Figure 1).

### 2.4. Sequencing of the CAPN3 Gene

Mutations in *CAPN3* were screened through direct sequencing by using primer pairs for the 24 coding *CAPN3* exons, GCF_000001405.25_GRCh37.p13 (19 April 2017). All exons were amplified with PCR by using a hot start Taq polymerase (Qiagen). PCR products were sequenced by using the BigDye Terminator Cycle Sequencing kit (Applied Biosystems). Amplicons were analyzed with capillary electrophoresis on an automated sequencer 3130 (Applied Biosystems), and the obtained DNA sequences were manually compared with wild type gene sequences.

## 3. Results

There are five living patients with the phenotype—two males and three females (Figure 1). The ages of the onset of the symptoms were eight (case 1, female), 14 (case 2, male), 10 (case 3, female), 12 (case 4, male), and 10 (case 5, female) with an average age of onset of 10.8 years and their ages at the moment of the last clinical exam being 14, 22, 29, 41 and 44, respectively (Table 1).

For all of the patients, the parents described toe walking as the first clinical manifestation. During the early phase of the disease (functional stage II, case 1), bilateral contractures of the elbows, ankles, and Achilles tendons, all of which appeared with the shortening of the calf muscles. Furthermore, the bilateral and symmetrical weakness of the pelvic girdle and the inferior members, while the impossibility of the abduction or extension of the legs and the areflexia of the lower extremities, appeared simultaneously.

At an average of 2.5 years later, weakness in the scapular girdle (functional stage lll, case 1) became evident with the presence of a winged scapula and areflexia in the superior extremities. In this phase, the onset of spinal rigidity, lumbar lordosis, and generalized primary contractures in the elbows, shoulders, knees, and hips were clearly observed, along with decreased cervical spine flexion due to contractures of the posterior cervical muscles.

**Figure 2:**
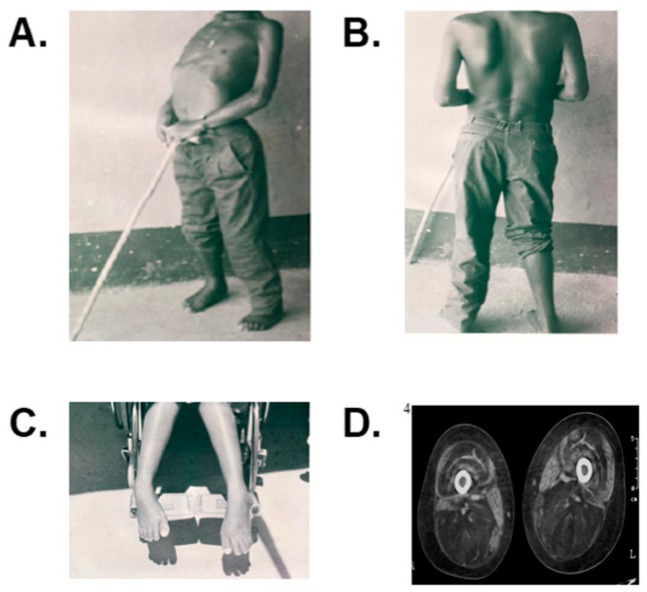
(**A**). Presence of severe generalized primary contractures of the elbows and lordosis shown in case 4, functional stage VI. (**B**). Winging scapula in case 4. (**C**). Presence of bilateral equinovarus foot in case 5, functional stage VII. (**D**). Case 4, functional stage VI, showing Mercuri grade IV affection with thigh proximal femur’s bilateral atrophy and a confluence of fatty infiltrated areas in the posterior, anterior, and the adductors muscular groups with unaffected gracilis and sartorius muscles.

The advanced stages were characterized by a complete inability to climb stairs (stage lV, case 2) or to stand up from a sitting position (stage V, case 3). By functional stage VI (case 4), walking could only be done with assistance (Figure 2). At this stage, bilateral talipes equinovarus (club foot) was clearly evident. In functional stage VII, i.e., case 5, the patient was confined to a wheel chair (Figure 2) with demonstrable abdominal laxity and curled fingers that were not present in the other patients. The involvement of the facial muscles was not observed. We closely evaluated each of the following functional stages: II, III, IV, V, VI, and VII. Likewise, computerized axial tomography made it possible to distinguish the pattern of muscular involvement of the case 4 lower limbs that were classified as part of functional stage VI and case 5 that was classified as functional stage VII, and both patients were categorized as Mercuri grade IV (severe) with more than 60% of muscle involvement (see Figure 2) [11]. The cardiac evaluation did not reveal any abnormalities. The electrocardiogram and echocardiogram results were normal for all of the patients. For the patient in functional state VI (case 4) and the patient in functional stage VII (case 5), a decrease in the diaphragmatic function was revealed by spirometry, with a reduction in vital capacity of up to 35% and 55%, respectively, for each patient. The chest X-rays of these two patients revealed a flattened hemidiaphragm.

**Figure 3:**
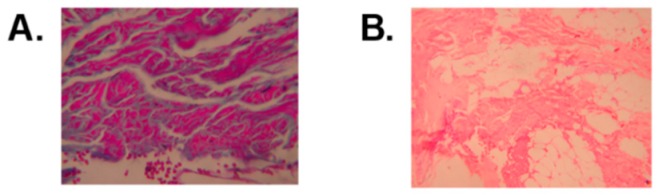
(**A**). Mason’s trichromic stain histopathological view that reveals hypertrophy, atrophy and normal striated muscle fibers that are surrounded by fibrosis and perimysial fat; magnification 10×. (**B**). Hematoxylin–eosin stain that shows striated muscle with intermingled hypertrophic, atrophic, and normal fibers that are surrounded by a proliferation of collagenous fibro connective tissue that is replaced, in other instances, by adipose tissue; magnification 4×.

Serum CK levels were as high as 15 times the normal reference value for the patient in functional states II–III, and they were twice the normal value in the functional state VII patient. In all five patients, the sensory and motor neuroconduction velocities demonstrated latencies, increased amplitudes of potentials, and normal velocities of conduction. Electromyography studies revealed a myopathic pattern. The histopathological cuts with hematoxylin–eosin and Mason’s trichromic stains revealed a muscular dystrophy pattern (Figure 3). The psychometric evaluations that were performed on all the patients disclosed an average intelligence quotient of 79 (normal range 61–94).

**Figure 4:**
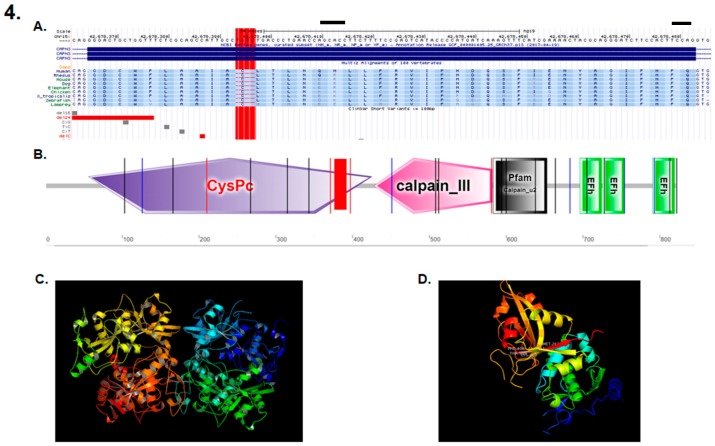
(**A**) Localization of the mutation consisting of a DNA deletion of four bases (TGCC) in exon 3 of the *CAPN3* gene (409–412 del TGCC) (red rectangle). (**B**) The mutation is located within the calpain-like thiol protease family (peptidase family C2) region, with the large subunit corresponding to a calcium activated neutral protease. (**C**) Main calpain 3 protein. (**D**) Domain I of the calpain 3 protein when the described mutation is present.

DNA sequencing revealed a novel deletion of four bases (TGCC) in exon 3 of the *calpain 3* gene (*CAPN3*) (NM_000070.2; NP_000061.1) (g.409_412del), (Figure 4A) that was homozygous in the five affected patients and was heterozygous in the two parents. The specific mutation site occurred at the protein CysPc domain (Figure 4B), triggering a change from the standard protein structure (Figure 4C) to a modified truncated protein structure when the mutation was present, thus affecting motifs within the calpain-like thiol protease family (peptidase family C2) region, with the large subunit corresponding to a calcium-activated neutral protease. (Figure 4D). The linkage analysis with microsatellite markers spanning the *CAPN3* gene reported a significant linkage to the gene, thus suggesting a founder effect that was inferred by the rareness and novelty of the mutation. 

## 4. Discussion

The laboratory and clinical analyses carried out for this study led to the diagnosis of calpainopathy for the five patients. Besides presenting the classical characteristics described in previously published studies, these patients also presented generalized primary contractures and spinal rigidity in the early stages of the disease, as well as bilateral clubfoot in the most affected patients, with the selective involvement of certain distal muscles in the extremities. This severe “contracted” phenotype was described as being similar to an Emery–Dreifuss-like syndrome due to the presence of early bilateral contractions in the neck, elbows, ankles, and Achilles tendons.

Pollitt et al. already showed the presence of generalized primary contractures, spinal rigidity, and distal weakness in a group of patients from Britain [10]. Mercuri et al. reported the development of early contractures including fingers’ flexors in patients from Britain [11], similar to those reported in these consanguineous Amerindian patients from Colombia. These findings are important for the clinical findings’ correlation to the very severe “contracted” phenotype and the range of associated mutations. All the patients presented a novel deletion of four bases (TGCC) in exon 3 of the calpain 3 gene (*CAPN3*) (NM_000070.2; NP_000061.1) (g.409_412del), thus leading to a change in the reading frame of this gene and to a truncated protein. 

Though the precise role of the calpain family is still poorly understood, calpains have been shown to be active in the proteolysis of a variety of substrates and to participate in the cell mobility, cell cycle and cell fusion of myoblasts [15]. We hypothesized that the 409–412 del TGCC in *CAPN3* could affect the calcium binding capacity, as structural studies have revealed that calpain’s Ca^2+^-requiring protease activity is mediated by Ca2+ binding to the CysPc domain, which is located in the calpain-like thiol protease family (peptidase family C2) domain, and amino acids 56–425 [15,16,17]. Furthermore, it seems that the 409–412 del TGCC in *CAPN3* may affect calcium-dependent proteolytic system and muscle physiology, as shown by the calpain 3-related, limb girdle muscular dystrophy R1 symptomatology. It was shown that calpain cleavage of desmin, talin, myristoylated alanine-rich C kinase substrate (MARCKS) actin binding and fibronectin caused membrane fluidity and cytoskeletal organization, thereby allowing myoblasts to fuse [18,19]. Calpain 3-related, LGMDR1 was not affected during embryogenesis, suggesting that calpain plays a role later in life, during myogenesis and sarcomere remodeling. In that vein, calpain 3 is able to cleave many cytoskeletal proteins and intervene in muscle–cell–cytoskeleton regulation, particularly during processes such as adaptive responses to exercise or regeneration after muscle wasting. Additionally, evidence has suggested that the inhibition of calpains leads to a reduction of myoblast migration in cell cultures [18], which could be linked to the control of cell membrane proteolysis and its requirement for cell migration, an earlier event for cell adhesion and de-adhesion [20]. We believe that the lack of production of a functional protein would cause a more severe phenotype than that found in other populations. Additionally, the patients in this study are the first Amerindians reported with this disease, and we cannot discard that the phenotypical differences of this syndrome may be related to the genetic background of the patients.

The community described in this study has a high consanguinity and has remained geographically isolated from other populations due to the surrounding mountains. The mutation described herein has not been reported in other studies, which suggests the existence of a founder effect in this particular population.

In light of our findings and with the antecedent that Pollitt et al. [10] and Mercuri et al. [11] established a differential diagnosis with Emery–Dreifuss syndrome, we recommend mandatory screening for calpainopathy in all patients who present an Emery–Dreifuss-like muscular dystrophy or those presenting a non-congenital illness with early primary contractures and who, because of other data, are suspected of having muscular dystrophy.

## 5. Conclusions

We report here findings from a Colombian pedigree with calpain 3-related, limb-girdle muscular dystrophy type r1 born from parents with known consanguinity, being the first Amerindian family reported with this disorder. The molecular diagnosis showed a frameshift mutation of a novel deletion of four base pairs that might explain the severe contracted phenotype observed in these patients and it would help to the outlining of this calpainopathy phenotype.

## Figures and Tables

**Table 1:** Clinical data on Colombian calpain 3-related LGMD R1 patients. Cases are referenced as in the genealogical tree (Figure 1).

Case	Sex	Age at Onset (Years)	Onset of Impairment	Age	Functional Stage	Age Ambulation Lost (Years)	CK	Observations
V3	Female	8	Pelvic	14	II–III	Still walking	X 15	Contractures of the elbows, ankles neck and Achilles tendons
V4	Male	14	Pelvic	22	IV	Still walking	X 14	Described clinical manifestations for case 1 plus inability to climb stairs
V5	Female	10	Pelvic	29	V	Still walking	X 10	Described manifestations for cases 1 and 2 plus inability to stand up from a sitting position
V7	Male	12	Pelvic	41	VI	Walks only with aid	X 4	Described above for cases 1, 2 and 3 plus walking only with assistance and equinovarus foot.
V8	Female	10	Pelvic	44	VII	Ambulation lost	X 2	Manifestations above plus ambulation lost
